# Heterosexual Practices Among Young People in Britain: Evidence From Three National Surveys of Sexual Attitudes and Lifestyles

**DOI:** 10.1016/j.jadohealth.2017.07.004

**Published:** 2017-12

**Authors:** Ruth Lewis, Clare Tanton, Catherine H. Mercer, Kirstin R. Mitchell, Melissa Palmer, Wendy Macdowall, Kaye Wellings

**Affiliations:** a Centre for Sexual and Reproductive Health Research, Faculty of Public Health and Policy, London School of Hygiene and Tropical Medicine, London, United Kingdom; b Centre for Sexual Health & HIV Research, Research Department of Infection and Population Health, University College London, London, United Kingdom; c MRC/CSO Social and Public Health Sciences Unit, Institute of Health and Wellbeing, University of Glasgow, Glasgow, United Kingdom

**Keywords:** Young people, Early adulthood, Heterosexual, Sexual behavior, Oral sex, Anal intercourse, Population survey

## Abstract

**Purpose:**

To describe time trends and current patterns in sexual practices with opposite-sex partners among men and women aged 16–24 years in Britain.

**Methods:**

Complex survey analyses of cross-sectional probability survey data from three British National Surveys of Sexual Attitudes and Lifestyles (Natsal), conducted approximately decennially 1990–2012, involving interviews with 45,199 people in total.

**Results:**

Birth cohort analysis showed a decline in the median age at first sexual experience and first intercourse since the midtwentieth century and a narrowing of the interval between these events. Comparison of data from Natsals 1, 2, and 3 showed increases in the prevalence of ever experience of oral and anal sex among 16- to 24-year-olds, which were more marked among older respondents in this age group between Natsals 1 and 2, and among younger respondents between Natsals 2 and 3. Among the sexually active, vaginal and oral sex remained the most common combination of practices reported in the past year. The proportion reporting a past-year repertoire of vaginal, oral, and anal sex rose from approximately one in 10 in 1990–1991 to approximately one in four men and one in five women in 2010–2012. In the latest survey, heterosexual experience of practices was positively associated with bisexual attraction among women.

**Conclusions:**

Recent decades have seen an earlier age at initiation of partnered sexual experiences and increased diversity in heterosexual practices among young people. Keeping pace with trends in sexual practices is necessary to safeguard young people's health and to support them in increasing their sexual well-being.

**Implications and Contribution**

This study uses nationally representative data from surveys of >45,000 people in Britain to document changes in heterosexual practices among young people. The earlier age at initiation of partnered sexual experiences and increased diversity of practices pose not only new challenges but also fresh opportunities for sexual health promotion.

A large body of empirical work has documented marked changes in the sexual behavior of young people over recent decades. Much of this research has focused on vaginal intercourse between men and women, especially the timing and circumstances surrounding its first occurrence. In Britain, for instance, studies have described a decline over time in median age at first intercourse, a narrowing of the gap between men and women in terms of timing, and increasing use of condoms at first sex [1], [2], [3].

Less empirical attention has been paid to sexual practices other than vaginal intercourse, despite evidence of upward trends in oral and anal sex among young people in several countries [4], [5], [6], [7], [8], [9]. Recently, however, researchers in the United States have used nationally representative data to gain a fuller picture of patterns of emerging heterosexual behavior among young people, and how they vary by sociodemographic characteristics. For instance, analyses of data from wave IV respondents to the National Longitudinal Study of Adolescent Health (Add Health) reveal striking gender differences in the order in which different practices are initiated, with women more likely than men to initiate vaginal intercourse before other practices and less likely to experience a second new behavior within the same year [10]. Other analyses of this data have also shown the co-occurrence of different practices within adolescents' repertoires of experience to vary by gender. While vaginal and oral-genital contact was the most common combination of sexual practices experienced by age 18 years for both men and women, the next most common combination for men was oral, vaginal, and anal sex, whereas for women it was vaginal intercourse only [11]. Despite the value of gaining a more comprehensive understanding of patterns of sexual behavior among young people, in Britain, analyses of data from population surveys have rarely examined aspects other than the prevalence of discrete sexual practices. Little is known about the average age of first sexual experience relative to first intercourse, nor the combinations of practices that co-occur within repertoires of experience, nor how such patterns are shifting over time.

Documenting changing patterns of emerging sexual behavior is important for several reasons. First and foremost, description of a range of sexual practices and the relationships between them aligns with calls for research that conceptualizes the initiation of sexual activity as a normative developmental process, rather than the conventional framing as “problematic [10], [12].” In addition, basic description of behavioral trends also provides important empirical context for examining associations between patterns of emerging sexual behavior and aspects of sexual health and well-being among young people. Such associations have not yet, to our knowledge, been explored within British studies, though research from other high-income countries has found associations between certain patterns of initiation (characterized by factors such as the relative timing, spacing, and sequencing of different sexual activities) and measures of sexual risk behavior and unintended outcomes, including sexually transmitted infection diagnosis, concurrent partnerships, and, among women, unprotected anal intercourse with their most recent partner [13], [14]. Beyond dimensions of sexual risk, however, little is known about whether or how patterns of emerging behavior are associated with aspects of sexual experience that foster sexual health and well-being. Research indicates repertoires of practice within a specific encounter can have implications for the quality of a sexual interaction; among women, for instance, receiving manual and/or oral stimulation has been associated with a greater likelihood of achieving orgasm [15], [16]. Thus, documentation of a wider range of practices may also lay the empirical foundation for examination of factors associated with sexual pleasure, satisfaction and well-being among young people. Gaining a more complete picture of sexual practices among young people is important, too, for informing efforts to improve sexual health. Population surveys in the United States [17], for example, have shown oral and anal sex with an opposite-sex partner is more commonly reported by women reporting bisexual attraction than those reporting heterosexual attraction only, which prompts the need for more nuanced targeting.

In this article, we report analyses of data from three British National Surveys of Sexual Attitudes and Lifestyles (Natsal), conducted approximately decennially between 1990 and 2010, with the aim of examining heterosexual experience of practices among young men and women aged 16–24 years. We restricted the analysis to examine practices between men and women only since the number reporting same-sex experience did not allow for detailed analysis. We use data from Natsals 1–3 to examine trends in young people's heterosexual practices in recent decades, including (1) relative timing of first sexual experience and first intercourse; (2) changes in the prevalence of ever experience of different sexual practices; and (3) changes in the combinations of sexual practices engaged in by young people. In addition, we use data from the most recent survey (Natsal-3), to describe patterns of heterosexual behavior among young people, including (4) recency with which different sexual practices feature in their sexual repertoires; and (5) variation in experience of heterosexual practices by reported sexual identity and sexual attraction.

## Methods

### Data and sample

Natsal surveys were carried out in 1990–1991 (Natsal-1), 1999–2001 (Natsal-2), and 2010–2012 (Natsal-3). In all three surveys, stratified random probability sampling was used to select households, from which one eligible individual, resident in Britain, was selected at random and invited to participate. Similar measures and procedures were used for all three surveys. In Natsal-1, pen-and-paper was used for both the face-to-face and self-completion interviews, with a self-completion booklet for more sensitive questions. In Natsals 2 and 3, participants were interviewed using computer-assisted personal interviewing (CAPI) with computer-assisted self-interview (CASI) for the more sensitive questions. Full details of the methodology are published elsewhere [18], [19], [20]. Natsal-3 was granted ethical approval by the Oxfordshire Research Ethics Committee A (reference: 09/H0604/27).

In Natsal-1, 18,876 men and women aged 16–59 years were interviewed (1,489 men and 1,888 women aged 16–24 years); the respective figures for Natsal-2 were 11,161 men and women aged 16–44 years (1,231 men and 1,442 women aged 16–24 years) and for Natsal-3 15,162 men and women aged 16–74 years (1,729 men and 2,140 women aged 16–24 years). The overall response rate was 66.8% for Natsal-1, 65.4% for Natsal-2, and 57.7% for Natsal-3 (64.8% among Natsal-3 participants aged 16–34 years).

### Measures

#### Sexual practices with an opposite-sex partner

Variables relating to first sexual experience and first intercourse with an opposite-sex partner were derived from answers to questions asked in the face-to-face (CAPI) section of the questionnaire. All participants were given a show card asking: “How old were you when you first had sexual intercourse with someone of the opposite sex, or hasn't this happened?” and “How old were you when you first had any type of experience of a sexual kind—for example kissing, petting, or feeling one another—with someone of the opposite sex (or hasn't this happened either)?” and were asked to give their age at these events, or to say that it had not happened. Those reporting intercourse having occurred before age 13 years were then asked “Has this happened with anyone else since you turned 13?” Intercourse was not defined in the CAPI section of the questionnaire.

Variables relating to other sexual practices were derived from answers to questions asking about the most recent experience of specific sexual acts with an opposite-sex partner, that is, vaginal intercourse, giving oral sex to a partner, receiving oral sex from a partner, anal intercourse, and genital contact not leading to intercourse (vaginal, oral, or anal). These questions were asked in the self-completion section of the survey (CASI) of participants who reported any sexual experience (and those who declined to answer the question about sexual experience). Response options were: in the last 7 days; between 7 days and 4 weeks ago; between 4 weeks and 6 months ago; between 6 months and 1 year ago; between 1 year and 5 years ago; longer than 5 years ago; never.

#### Sexual attraction and identity

Variables relating to sexual attraction and identity were derived from questions asked in the CAPI section of the questionnaire, following the questions on first intercourse and first sexual experience. For sexual attraction, participants were handed a show card with the statement “I have felt attracted…” and asked to give the letter corresponding to their choice of response option from a list comprising: only to females, never to males; more often to females, and at least once to a male; about equally often to females and to males; more often to males, and at least once to a female; only ever to males, never to females; I have never felt sexually attracted to anyone at all (response options for men given as an example).

The question on sexual identity was asked face-to-face after the CASI section of the questionnaire (only in Natsal-3), when participants were handed a show card with the question ‘Which of the options on this card best describes how you think of yourself?’ and asked to give the letter corresponding to their answer from response options: heterosexual/straight; gay/lesbian; bisexual; other.

### Statistical analysis

We performed all analyses using the survey commands in Stata (v13.1), which account for the weighting, clustering, and stratification of the data. We carried out survival analysis of data from participants aged 16–74 years in the most recent survey (Natsal-3) to describe trends in median age at first sexual experience, and in first intercourse, with an opposite-sex partner by successive 5-year birth cohorts, plotting the lines representing these trends on the same graph to show changes in the interval between them. Using data from all three Natsals, we estimated the proportion of young people reporting ever experience of each sexual practice with an opposite-sex partner in each of 3-year age groups (16–18, 19–21, and 22–24 years), and we present odds ratios (ORs) and 95% confidence intervals to show changes between surveys, using Natsal-2 as the baseline. To examine how the repertoire of practices experienced in the past year has changed over the three surveys, we used proportional Venn diagrams to show the proportions of young people aged 16–24 years reporting different combinations of practices (vaginal intercourse, any type of oral sex, and/or anal sex) in the past year, among those reporting at least one of these practices in the same time period.

To describe current patterns of sexual practices we used data from Natsal-3 to estimate the proportion of young men and women who reported each practice in different time periods (past year, past month, and past week), as a proportion of those reporting ever experience of that practice. Finally, using data from Natsal-3, we estimated the prevalence of ever experience of specific practices with an opposite-sex partner by sexual attraction and identity among 16- to 24-year-olds. Owing to small numbers, we excluded individuals who reported no sexual attraction (17 men and 30 women) and those who reported being attracted only to people of the same sex (15 men and 12 women). Ranges for missing data for the five sexual practices examined are as follows: 2%–6% in Natsal-1, 2%–5% in Natsal-2, and <3% in Natsal-3. Missing data for variables on sexual identity and attraction in Natsal-3 were <.5%.

## Results

### Trends in the timing of first heterosexual experience and first intercourse

**Figure 1:**
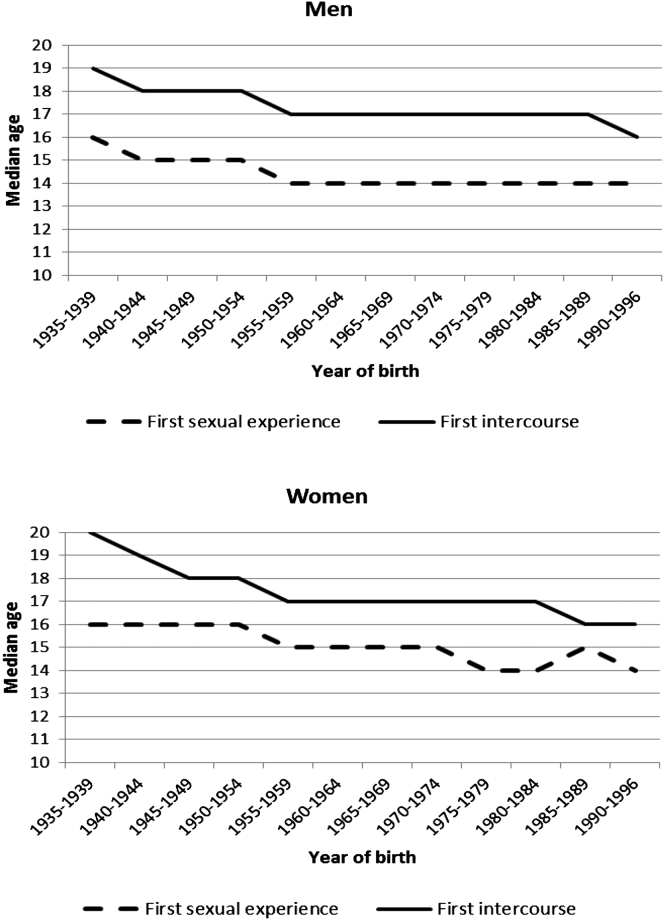
Median age at first sexual experience and first intercourse by birth cohort.

Figure 1 shows trends in median age at first sexual experience with an opposite-sex partner plotted alongside trends in median age at first intercourse, by successive birth cohorts within Natsal-3. For both men and women, median age at first heterosexual experience has declined since the midtwentieth century, from 16 among participants in the earliest birth cohort (born 1935–1939) to 14 in the most recent birth cohort (born 1990–1996). Median age at first intercourse has declined from 20 for women and 19 for men, born in the late 1930s to 16 for both men and women born in the early 1990s. Among both men and women, the interval between median age at first sexual experience and first intercourse has narrowed over time, from 4 years among women and 3 years among men born in the late 1930s to 2 years for those born in 1990–1996.

### Trends in sexual practices with an opposite-sex partner

Table 1 compares the prevalence of sexual practices among participants in different age ranges within the sample aged 16–24 years between Natsals 1, 2,a and 3.

**Table 1:** Ever reporting of practices with an opposite-sex partner by young people aged 16–24 years in Natsals 1–3

	16–18			19–21			22–24			16–24		
	Natsal-1	Natsal-2	Natsal-3	Natsal-1	Natsal-2	Natsal-3	Natsal-1	Natsal-2	Natsal-3	Natsal-1	Natsal-2	Natsal-3
Men												
Vaginal intercourse												
%	55.1	57.6	61.4	84.7	90.8	84.9	92.2	93.1	90.2	78.6	79.2	79.0
OR (95% CI)	.90 (.66–1.23)	1	1.17 (.88–1.55)	**.56 (.35–.90)**	1	**.57 (.36–.90)**	.88 (.51–1.50)	1	.68 (.40–1.16)	.96 (.78–1.18)	1	.99 (.81–1.21)
Oral-penis contact												
%	40.7	48.0	57.2	71.5	84.3	79.9	81.0	86.9	86.1	65.8	71.7	74.6
OR (95% CI)	.75 (.55–1.02)	1	**1.45 (1.09–1.92)**	**.47 (.31–.71)**	1	.74 (.48–1.13)	**.64 (.43–.96)**	1	.93 (.60–1.44)	**.76 (.63–.92)**	1	1.16 (.95–1.40)
Oral-vulva contact												
%	40.6	46.5	51.1	72.8	79.5	75.8	82.2	86.3	84.6	66.6	69.4	70.6
OR (95% CI)	.78 (.58–1.07)	1	1.20 (.91–1.58)	.69 (.46–1.03)	1	.81 (.54–1.21)	.73 (.49–1.09)	1	.87 (.58–1.32)	.88 (.73–1.06)	1	1.06 (.88–1.28)
Anal intercourse												
%	7.2	8.1	14.3	10.3	20.9	33.5	13.5	25.6	39.4	10.5	17.6	29.2
OR (95% CI)	.87 (.49–1.54)	1	**1.89 (1.21–2.95)**	**.43 (.28–.66)**	1	**1.91 (1.37–2.66)**	**.45 (.31–.65)**	1	**1.89 (1.36–2.62)**	**.55 (.42–.70)**	1	**1.92 (1.57–2.36)**
Genital contact												
%	69.3	81.9	76.4	85.8	87.9	85.0	87.3	90.6	87.9	82.5	87.2	83.6
OR (95% CI)	**.50 (.33–.76)**	1	.71 (.48–1.05)	.83 (.52–1.32)	1	.77 (.50–1.21)	.71 (.45–1.14)	1	.76 (.46–1.24)	**.69 (.53–.91)**	1	**.75 (.58–.97)**
Women												
Vaginal intercourse												
%	52.9	57.4	60.4	87.5	91.9	85.2	94.7	95.8	89.3	79.8	81.2	78.8
OR (95% CI)	.84 (.63–1.10)	1	1.14 (.87–1.49)	**.62 (.39–.99)**	1	**.51 (.31–.83)**	.79 (.43–1.45)	1	**.36 (.21–.62)**	.91 (.75–1.12)	1	.86 (.70–1.05)
Oral-penis contact												
%	39.6	48.6	55.9	70.1	82.5	77.1	77.9	89.0	84.1	63.8	72.9	72.9
OR (95% CI)	**.69 (.52–.93)**	1	**1.34 (1.02–1.77)**	**.50 (.35–.69)**	1	.71 (.50–1.01)	**.44 (.30–.63)**	1	**.65 (.45–.96)**	**.65 (.55–.78)**	1	1.00 (.83–1.20)
Oral-vulva contact												
%	41.8	49.0	53.6	74.0	84.0	77.8	79.2	89.7	82.7	66.2	73.8	71.9
OR (95% CI)	.75 (.56–1.00)	1	1.20 (.91–1.58)	**.54 (.39–.76)**	1	**.67 (.47–.95)**	**.44 (.30–.63)**	1	**.55 (.37–.80)**	**.69 (.58–.83)**	1	.91 (.76–1.09)
Anal intercourse												
%	7.6	7.1	16.2	12.9	21.5	34.3	16.5	27.0	34.2	12.5	18.3	28.5
OR (95% CI)	1.06 (.62–1.82)	1	**2.52 (1.61–3.94)**	**.54 (.38–.77)**	1	**1.91 (1.40–2.61)**	**.53 (.39–.73)**	1	**1.40 (1.07–1.85)**	**.64 (.52–.80)**	1	**1.78 (1.48–2.15)**
Genital contact												
%	76.3	82.2	78.7	82.0	91.3	83.9	87.3	92.3	84.1	82.8	89.2	82.6
OR (95% CI)	.69 (.45–1.08)	1	.80 (.52–1.21)	**.43 (.29–.64)**	1	**.49 (.33–.75)**	**.57 (.37–.90)**	1	**.44 (.28–.68)**	**.58 (.45–.76)**	1	**.57 (.44–.74)**
Denominator^a^ (unweighted, weighted)												
Men	384, 606	445, 554	656, 394	480, 733	365, 466	565, 451	560, 724	390, 475	492, 382	1,424, 2,063	1,200, 1,495	1,713, 1,227
Women	484, 587	465, 514	758, 381	619, 707	483, 522	631, 378	707, 693	477, 461	729, 435	1,810, 1,987	1,425, 1,496	2,118, 1,195

ORs (95% CI) where 95% CI does not cross 1 are in bold.

CI = confidence interval; OR = odds ratio.

a Denominators shown are for vaginal intercourse; denominators for other practices vary slightly. Denominators for genital contact in 16- to 18-year-olds differ, these are: men: 268, 420 (Natsal-1); 298, 369 (Natsal-2); and 511, 313 (Natsal-3); women: 323, 376 (Natsal-1); 314, 352 (Natsal-2); and 591, 298 (Natsal-3).

Unsurprisingly, the prevalence of ever experience of the sexual practices increased with age in all three surveys. Looking at the pattern in the latest survey (Natsal-3), for example, the practice most commonly reported by the youngest participants (aged 16–18 years) was genital contact not leading to intercourse (76.4% men and 78.7% women). Three out of five of those aged 16–18 years had had vaginal intercourse, and over half reported experience of each type of oral-genital contact. A slightly smaller proportion of men than women in this age group reported experience of anal intercourse (14.3% of men and 16.2% of women). By age 22–24 years, approximately nine out of 10 reported experience of vaginal intercourse (90.2% men and 89.3% women), and over four out of five reported experience of oral-penis contact (86.1% men and 84.1% women), oral-vulva contact (84.6% men and 82.7% women), and genital contact (87.9% men and 84.1% women). Over a third of those aged 22–24 years reported experience of anal intercourse (39.4% men and 34.2% women).

Between Natsals 1 and 2, reporting of sexual practices increased for both men and women in all three age groups. Changes seen between Natsals 2 and 3, however, were less consistent, especially among young women. Comparing Natsal-3 with Natsal-2, we observe the most marked increases in the youngest men and women (16–18 years), including increased reporting of oral-penis contact (ORs: 1.45 men, 1.34 women) and anal intercourse (ORs: 1.89 men, 2.52 women). Conversely, among women in the two older age groups (19–21 and 22–24 years), we observe significant decreases in reporting of all practices between Natsals 2 and 3, with the exception of anal sex. Among men aged 19–21 years, the proportion reporting vaginal intercourse also decreased between Natsals 2 and 3 (OR: .57). The most marked change between Natsals 2 and 3 was the significant increase in anal sex among both men and women across all age groups. Over the time span of the three surveys, the proportion of women aged 22–24 years reporting having ever experienced anal sex more than doubled, from 16.5% to 34.2%, and among men of the same age showed an almost threefold increase, from 13.5% to 39.4%.

### Trends in repertoires of heterosexual practice

**Figure 2:**
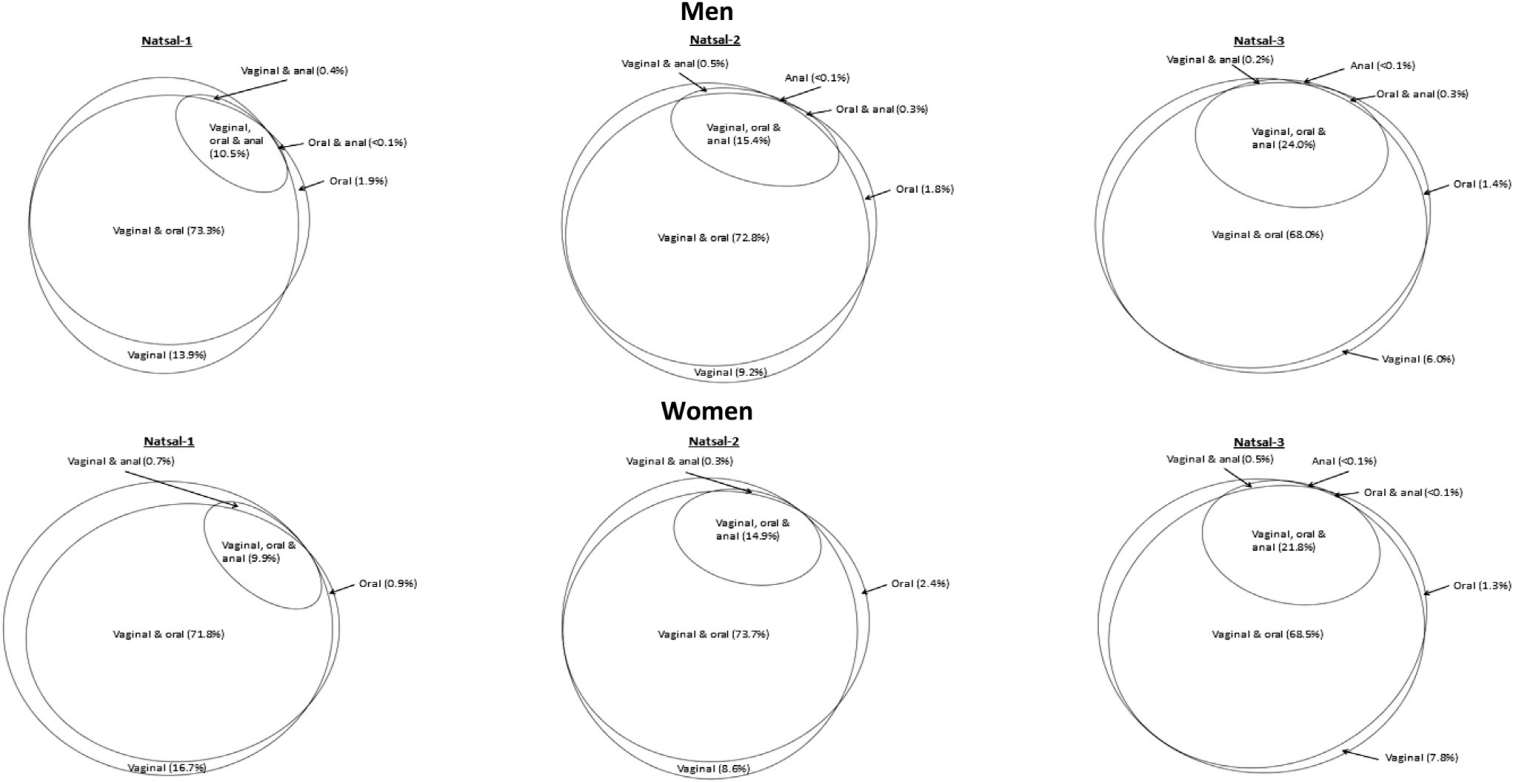
Reporting of vaginal intercourse, oral-genital contact (any), and anal intercourse in the past year proportionally for 16- to 24-year-old men and women in Natsals 1–3. Denominator: those reporting one or more practice (vaginal, oral, or anal) in the past year.

Figure 2 represents changes in the combinations of sexual practices reported by those aged 16–24 years in each Natsal survey, among those reporting experience of at least one of the practices (vaginal intercourse, oral sex (oral-penis and/or oral-vulva), and anal intercourse), in the past year. Across all three surveys, vaginal intercourse and oral sex was the most common combination of practices experienced in the past year, being consistently reported by more than two thirds of men and women, though the proportion declined between Natsals 2 and 3 from 72.8% to 68.0% among men (*p* = .041) and 73.7%–68.5% among women (*p* = .007). However, the proportion of sexually active 16- to 24-year-olds who had experienced oral, vaginal, and anal sex in the past year increased with each survey, from 10.5% of men and 9.9% of women in Natsal-1, to 24.0% of men and 21.8% of women (*p* < .0001 in both cases) in Natsal-3, making this the second most common combination of practices among both men and women in Natsals 2 and 3. Between Natsal-1 and Natsal-3, there has been a concomitant decrease in the proportion who have only experienced vaginal intercourse and no other practices in the past year, from 13.9% to 6.0% among men and from 16.7% to 7.8% among women (*p* < .0001 in both cases). The proportion of men and women reporting repertoires of only vaginal and anal intercourse, or only oral sex and anal intercourse, in the past year was negligible (<1%) across all three surveys.

### Recency of sexual practices with an opposite-sex partner

**Figure 3:**
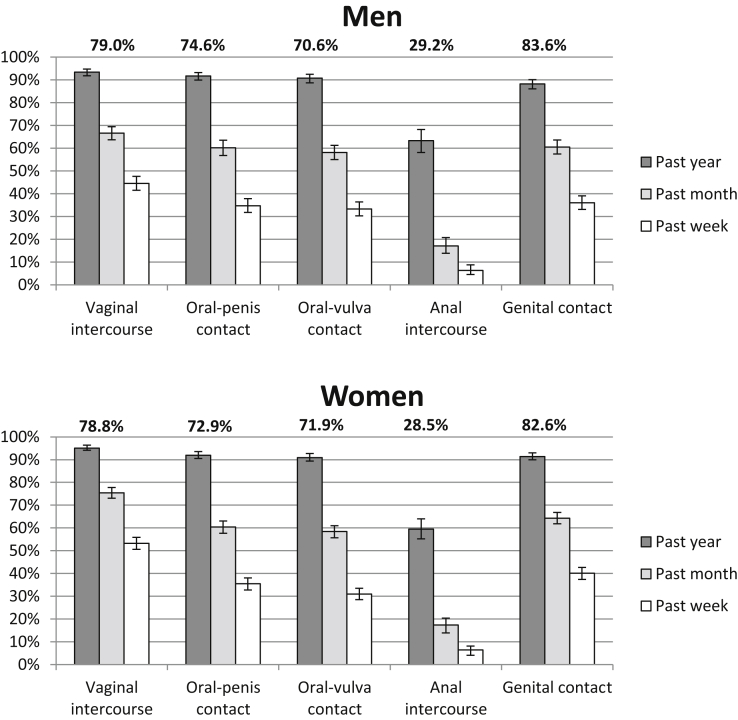
Recency of occurrence of practices with an opposite-sex partner, among 16- to 24-year-olds in Natsal-3 who report ever experience of that practice with an opposite-sex partner. Percentages above bars indicate percentage of people reporting ever experience of each practice. Denominators: those who report ever experience of a practice (unwt, wt): vaginal intercourse (M:1322, 970; W:1689, 941); oral-penis contact (M: 1244, 915; W:1566, 871); oral-vulva contact (M: 1178, 866; W: 1548, 859); anal intercourse (M: 488, 358; W: 618, 341); and genital contact (1307, 958; W: 1642, 918).

Figure 3 presents data on the recency of the occurrence of specific practices among 16- to 24-year-olds reporting ever experience of each practice. Vaginal intercourse, oral sex, and genital contact were all experienced within the last year by more than nine out of 10 men and women with ever experience of each practice and anal intercourse by three out of five (63.3% men and 59.5% women). More recent experience was somewhat lower in frequency. In the case of vaginal intercourse, the proportion of ever-experienced women reporting occurrence in the last month fell to three quarters, and in the last week to just over half, and the respective proportions among men were slightly lower. Compared with vaginal intercourse, the equivalent prevalences for oral sex and genital contact in the more recent time periods were slightly lower. Recent experience of anal sex was appreciably less common than other practices. Among participants with ever experience, the prevalence of occurrence fell to 17% in the past month and 6% in the past week.

### Heterosexual experience by sexual attraction and sexual identity

Table 2 presents prevalence of ever experience of sexual practices with an opposite-sex partner by self-reported sexual attraction and sexual identity, among men and women aged 16–24 years in Natsal-3. Among women, there was a clear association between reported sexual attraction and heterosexual experience of practices, with experience of each practice being more common among women who reported attraction to both men and women than among those who reported attraction only to men. Conversely, men who reported attraction only to women were more likely to have experienced each of the sexual practices with a woman than men who reported bisexual attraction (though the corresponding numbers are small and of borderline statistical significance).

**Table 2:** Variation in ever-experience of practices with an opposite-sex partner by self-reported sexual identity and attraction in 16- to 24-year-olds in Natsal-3

	Men						Women					
	%	95% CI	OR	95% CI	*p* value	Denom. (unwt., wt.)	%	95% CI	OR	95% CI	*p* value	Denom. (unwt., wt.)
Vaginal intercourse												
Sexual attraction					.0888						<.0001	
Opposite sex only	80.6	78.3–82.7	1	—		1,567, 1,126	78.1	75.8–80.2	1	—		1,742, 966
Attracted to both opposite and same sex	73.5	63.8–81.3	.67	.42–1.06		114, 77	91.1	87.2–93.9	2.88	1.87–4.44		331, 199
Sexual identity					<.0001						.934	
Heterosexual/straight	80.2	77.9–82.3	1	—		1,652, 1,186	78.9	76.8–80.9	1	—		2,020, 1,132
Gay/lesbian/bisexual/other	45.2	31.7–59.5	0.20	.11–.36		60, 41	78.5	67.4–86.6	.98	.55–1.73		93, 60
Oral-penis contact												
Sexual attraction					.0952						<.0001	
Opposite sex only	76.3	73.8–78.6	1	—		1,566, 1,126	71.4	68.9–73.7	1	—		1,743, 967
Attracted to both opposite and same sex	68.3	57.7–77.3	.67	.42–1.07		115, 78	89.6	85.3–92.7	3.45	2.29–5.21		331, 199
Sexual identity					<.0001						.5365	
Heterosexual/straight	75.9	73.4–78.2	1	—		1,652, 1,186	73.2	70.9–75.3	1	—		2,021, 1,132
Gay/lesbian/bisexual/other	36.0	22.8–51.6	.18	.09–.34		60, 41	69.9	58.2–79.4	.85	.51–1.42		93, 60
Oral-vulva contact												
Sexual attraction					.2735						<.0001	
Opposite sex only	72.1	69.4–74.6	1	—		1,566, 1,126	70.2	67.7–72.5	1	—		1,743, 967
Attracted to both opposite and same sex	66.9	56.8–75.7	.78	.51–1.21		115, 78	89.0	84.6–92.3	3.44	2.30–5.16		331, 199
Sexual identity					<.0001						.7624	
Heterosexual/straight	71.8	69.2–74.2	1	—		1,652, 1,186	71.9	69.6–74.1	1	—		2,021, 1,132
Gay/lesbian/bisexual/other	36.8	24.4–51.3	.23	.13–.41		60, 41	73.5	62.1–82.5	1.09	.64–1.86		93, 60
Anal intercourse												
Sexual attraction					.1546						<.0001	
Opposite sex only	30.0	27.5–32.6	1	—		1,567, 1,126	26.7	24.5–29.0	1	—		1,745, 968
Attracted to both opposite and same sex	23.6	16.6–32.4	.72	.46–1.13		114, 77	41.0	35.3–46.9	1.91	1.47–2.48		331, 199
Sexual identity					.0165						.5192	
Heterosexual/straight	29.7	27.3–32.3	1	—		1,652, 1,186	28.4	26.1–30.7	1	—		2,023, 1,133
Gay/lesbian/bisexual/other	13.6	6.5–26.4	.37	.17–.84		60, 41	31.8	22.1–43.5	1.18	.71–1.96		93, 60
Genital contact												
Sexual attraction					.0916						<.0001	
Opposite sex only	85.5	83.2–87.5	1	—		1,423, 1,046	82.6	80.3–84.7	1	—		1,585, 887
Attracted to both opposite and same sex	78.8	69.0–86.1	.63	.37–1.08		111, 76	92.5	88.9–95.0	2.59	1.65–4.08		325, 197
Sexual identity					<.0001						.737	
Heterosexual/straight	84.6	82.4–86.6	1	—		1,507, 1,106	82.8	80.7–84.8	1	—		1,856, 1,049
Gay/lesbian/bisexual/other	56.2	42.4–69.1	.23	.13–.41		58, 39	81.3	71.1–88.5	.91	.51–1.62		92, 60

CI = confidence interval; OR = odds ratio.

With regards to sexual identity (as opposed to sexual attraction), a larger discrepancy is seen between men who identify as heterosexual and those who report a sexual identity other than heterosexual, with much lower odds of experiencing practices with an opposite-sex partner among the latter group (OR for vaginal intercourse: .20; 95% confidence interval: .11–.36). Among women, there were no significant associations between reported sexual identity and heterosexual experience of sexual practices.

## Discussion

These data from three decennial national probability surveys of more than 45,000 people document marked changes in heterosexual practices among young men and women in Britain. Birth cohort analyses of Natsal-3 data show a decline in median age at first sexual experience and first intercourse over the past half century and a narrowing of the interval between the two events, both of which trends are more marked among women than men. These trends likely reflect the relaxation of social attitudes regarding premarital sexual activity, in combination with young people's greater access to reliable contraception.

Comparison of data from Natsals 1, 2, and 3 reveals a widening of the range of sexual practices engaged in by 16- to 24-year-olds. The increasing prevalence of oral and anal sex was more noticeable among participants aged 19–24 years between Natsals 1 and 2 and among those aged 16–18 years between Natsals 2 and 3, such that trends in sexual practices which were initially observed among those in their late teens and early 20s appear to have filtered down the age range over time. Although ever experience of anal intercourse remains less commonly reported than the other practices measured, nevertheless, between Natsals 1 and 3 its prevalence among 16- to 24-year-olds almost tripled among men and more than doubled among women. These changes have been accompanied by modest declines in the prevalence of vaginal intercourse and genital contact.

Vaginal intercourse remains a mainstay of the heterosexual repertoire across all three surveys, having been practiced by all but a very small proportion (<2.5%) of young people who were sexually active in the year before interview. For the most part, then, we are seeing oral and anal sex joining, rather than replacing, vaginal intercourse in heterosexual repertoires. Moreover, despite the increasing prevalence of anal intercourse, the low prevalence of reporting recent experience may indicate it is a less regular feature in young people's sexual interactions.

Rising trends in oral and anal sex seen in our data have also been documented in population surveys in other high-income countries [21], [22] and can be interpreted in the context of a broader shift toward greater diversity in sexual behavior, including increases in reported numbers of sexual partners, and, among women, reporting of same-sex experience [1]. Factors driving the increasing diversity in sexual practices in particular are likely to be complex, such that single factor explanations should be treated with caution. For instance, while young people's increased access to pornography is routinely cited in discussion about increases in anal sex [23], empirical evidence of a causal relationship between exposure to explicit sexual media and sexual behavior practices remains equivocal [24], [25], [26], and young people's own accounts of anal sex reveal a complex socio-sexual landscape shaping practice, within which pornography is only one feature [33]. In addition, while there is a sizeable body of research examining predictors of specific sexual practices at the individual level [6], [26], [27], research is also needed examining factors operating at the macrolevel of sociocultural norms.

Strengths of this study include the use of nationally representative data ensuring generalizability of the findings; a large, boosted sample of young people enabling estimates of differences between groups and time periods to be made with confidence; and the inclusion of a variable relating to first sexual experience, rarely reported in behavioral research and usefully augmenting data on first intercourse to provide a more nuanced understanding of emerging sexual behavior.

Our study also has limitations. Despite the large sample size, the number of participants reporting same-sex experience was not sufficient to extend the analyses to practices between same-sex partners. We also rely on self-reports of behavior, which are likely to be influenced by prevailing social norms. As noted by others [5], it is difficult to assess the extent to which observed differences in the prevalence of sexual practices between time periods reflect real changes in behavior, or result from reporting biases attendant on shifts in social attitudes toward those practices. In addition, the scope of our analysis was limited by the fact that the surveys measured age at first sexual experience and first intercourse, but not that of other sexual practices, such that the relative timing and sequence of their initiation cannot be described. Furthermore, sexual identity and attraction were reported at the time of interview and may be subject to change over time. Finally, data collection for this study was completed toward the end of 2012, and further changes in young people's sexual practices may have occurred in the intervening period.

Our findings present both possibilities and challenges for public health policy and practice. The trend toward greater diversification in sexual behavior seen in this and other studies is further evidence of the fluidity of sexual behavior and of the human capacity for change in behavior in response to new influences. This potential for adaptation can be harnessed in efforts to improve sexual health and well-being, should modification be needed to adapt to new threats. At the same time, changing patterns of behavior raise new challenges for health promotion. Studies have shown, for example, lower use of condoms and higher user-error rates during anal intercourse between men and women [5], [6], [28], suggesting the need for efforts to encourage and advise on correct use. In addition, the higher prevalence of each sexual practice among women reporting bisexual attraction compared with women reporting attraction only to men suggests that efforts to promote sexual health and well-being of this group needs to take account of the complexities of interactions between identity, attraction, and behavior [29], [30].

Beyond a narrowly framed biomedical “risk perspective [31],” our findings also warrant attention to their relevance for sexual well-being among young people, including the quality and dynamics of their sexual interactions. In particular, the increasing diversity of repertoires of sexual practice must be considered in the context of evidence regarding gender disparities in expectations and experiences of these practices. Analysis of longitudinal data from the U.S. Add Health study has shown that repeated engagement in disliked sexual activities (mainly oral-penis contact and anal intercourse) was four times more common among women than men [32]. Evidence from our own qualitative research in England, too, has revealed prominent cultural discourses among teenagers that normalize painful, and sometimes coercive, anal intercourse [33], with men tending to talk more positively about the practice than women. Health care providers and educators need to understand the impact of gender dynamics on sexual practices in order to provide culturally sensitive and appropriate sex education emphasizing the importance of communication about sexual desires and concerns, and respecting and accepting a partner's dislike of, and possibly unwillingness to engage in, certain activities. Young people may also need guidance on how to communicate their preferences and dislikes [32]. As noted elsewhere [34], young people's sexual behavior is shaped by an ever-increasing number of influences; thus, keeping pace with trends in sexual practices is necessary to safeguard their health and to support them in increasing their sexual well-being.
